# Influence of age on postoperative complications especially pneumonia after gastrectomy for gastric cancer

**DOI:** 10.1186/s12893-019-0573-x

**Published:** 2019-08-08

**Authors:** Chikashi Shibata, Hitoshi Ogawa, Toru Nakano, Kaori Koyama, Kuniharu Yamamoto, Munenori Nagao, Daisuke Takeyama, Kazuhiro Takami, Akihiro Yasumoto, Tomohiko Sase, Shun-ichi Kimura, Kentaro Sawada, Yu Katayose

**Affiliations:** Division of Gastroenterologic and Hepato-biliary-pancreatic Surgery, Department of Surgery, Tohoku Medical and Pharmaceutical University, 1-12-1 Fukumuro, Miyagino-ku, Sendai, 983-8512 Japan

**Keywords:** Distal gastrectomy, Morbidity, Pneumonia, Total gastrectomy

## Abstract

**Background:**

The aim of this study was to investigate the influence of patients’ age on postoperative morbidities including pneumonia.

**Methods:**

We reviewed the clinical records of 211 patients with stages I – III gastric cancer undergoing curative distal gastrectomy (DG) or total gastrectomy (TG). Patients were classified into an elderly (≧80 y.o.) or a control (< 80 y.o.) group. We compared patient characteristics (sex ratio, disease stage, degree of lymph node dissection, number of retrieved lymph nodes, and type of reconstruction) and early postoperative outcomes (operation time, intra-operative blood loss, and postoperative morbidity including pneumonia, and mortality) between the two groups separately in DG and TG.

**Results:**

There were 134 and 77 patients who underwent DG and TG, respectively. The numbers of patients in the elderly and control groups were 25 and 109 in DG and 12 and 65 in TG. The percentage of female patients in the elderly group was greater than that in the control group in both DG and TG. The extent of lymph node dissection did not differ between two groups in TG; in contrast in DG, the rate of a D1 dissection was greater in the elderly group than in the control group. There were no differences between the two groups in distribution of disease stage, number of retrieved lymph nodes, operation time, and blood loss in DG and in TG. Overall postoperative morbidity did not differ between two groups after DG and after TG. The rate of infectious complications in the elderly group was not different from that in the control group after DG and after TG. The incidence of pneumonia was more frequent in the elderly group compared to the control group after DG (8% vs. 1%, *P* < 0.05) but not after TG (17% vs. 5%). When patients were compared between the elderly and the control groups regardless of type of gastrectomy, the incidence of pneumonia in the elderly group (4/37 (11%)) was greater than that in the control group (4/174 (2%), *P* < 0.05).

**Conclusions:**

These results suggest that pneumonia is increased in patients older than 80 years after DG.

## Background

In Japan, the mean expected life span is approximately 80 years in men and 87 years in women, and the percentage of elderly people in the total population is increasing [1]. Although the mortality from gastric cancer is decreasing, gastric cancer is still the 3rd most common cause of death due to malignant diseases in Japan [2]. As the prevalence of gastric cancer increases with age [2], it is likely that the number of elderly patients with gastric cancer requiring gastrectomy will not decrease but rather increase in a country like Japan.

Because the general medical condition of this elderly group is less robust, the percentage of patients who cannot undergo gastrectomy is considered high due to a greater prevalence of co-morbidities. Even though an extended lymphadenectomy during the gastrectomy was feasible, the degree of lymph node dissection in elderly patients is often less than that in young patients [3].

A considerable number of articles have been published in term of overall postoperative complications in elderly patients including postoperative pneumonia [3–10]. Some other studies investigated if age was a risk factor for postoperative complications or pneumonia [11–13]. It remains controversial, however, whether or not overall postoperative morbidity as well as pneumonia is increased after gastrectomy in elderly patients. The different definitions of ‘elderly’ patients in each study might be another confounding factor for these different results [3–10]. Few articles have studied postoperative complications in elderly patients after distal gastrectomy (DG) and total gastrectomy (TG) separately.

The aim of the present study was to investigate the influence of age on postoperative complications separately after the two most representative types of gastrectomy, DG and TG. We compared perioperative results including morbidity and pneumonia between elderly and control patients.

## Methods

We reviewed retrospectively the clinical records of the 211 patients who underwent DG and TG for stage I ~ III gastric cancer between 2008 and 2015. Of these, 134 and 77 underwent DG and TG, respectively. Patients with remnant gastric cancer were excluded, as were those who underwent non-curative resection, or concomitant resection of other organs due to synchronous malignancy other than gastric cancer, and those who had been treated with neoadjuvant chemotherapy,

Patients were divided into either the elderly group (≧80 years old) or the control group (< 80 years old) based on their age at the time of gastrectomy. We studied sex, pathologic stage of the gastric cancer, reconstruction method, degree of lymph node (LN) dissection and number of retrieved LNs as patient characteristics. The pathologic stage of the gastric cancer and the degree of LN dissection (D1, D1+, D2) were classified based on the Japanese classification of gastric carcinoma and the Gastric Cancer Treatment Guidelines in Japan [14, 15]. We also investigated operation time, intraoperative blood loss, and operative mortality and morbidity including pneumonia as early postoperative outcomes. Morbidity was defined as grades II-V according to Clavien-Dindo classification [16]. Patient characteristics and perioperative outcomes were compared between the two groups in those undergoing a DG and TG, separately. Pneumonia was diagnosed on imaging modalities such as plain chest X-ray or computed tomography scan in all patients, and complimentary laboratory data suggesting inflammation. Infectious complications included anastomotic leakage, intraabdominal abscess, pancreatic fistula, mediastinitis, pneumonia, urinary tract infection, and fever of unknown origin.

Values are shown in mean ± standard deviation. The Mann-Whitney U test was used for comparison of intraoperative blood loss, operation time, number of retrieved LNs, and postoperative hospital stay. The chi-square test was used to compare all parameters other than above. *P* values less than 0.05 were regarded as statistically significant.

## Results

Table 1 summarizes the patient characteristics and early postoperative outcomes when patients were divided into the elderly and control groups regardless of type of gastric resection. Average age was 83 ± 2 years in 37 elderly patients and 66 ± 9 years in 174 control patients. The ratio of female patients in the elderly group was greater than that in the control group (21/37 (57%) vs. 51/174 (29%), *P* < 0.05). There were no differences between the two groups in distribution of disease stage, DG:TG, the extent of LN dissection, the number of retrieved LNs, operation time, intraoperative blood loss, and the postoperative hospital stay. One patient died of postoperative complications in the elderly and the control groups, respectively. Overall morbidity did not differ between the two groups (27% vs. 18%), and the same was true for infectious complications (14% vs. 11%). The overall rate of postoperative pneumonia in the elderly group was greater than that in the control group (11% vs. 2%, *P* < 0.05). The rate of pneumonia in infectious complications was increased in the elderly group (4/5 (80%)) compared to that in the control group (4/20 (20%), *P* < 0.05) (Table 1).

**Table 1:** Patient characteristics in the elderly and control groups

	Elderly (*N* = 37)	Control (*N* = 174)
Age (Years)*	83 ± 2	66 ± 9
Male:Female*	16:21	123:51
Disease Stage		
I	19 (51%)	88 (50%)
II	6 (16%)	29 (17%)
III	12 (33%)	57 (33%)
DG:TG	12:25	65:109
LN Dissection		
D1	3 (8%)	3 (2%)
D1+	20 (54%)	85 (49%)
D2	14 (38%)	86 (49%)
Number of retrieved LNs	25 ± 12	28 ± 14
Operation Time (min)	232 ± 48	249 ± 51
Blood Loss (mL)	275 ± 278	310 ± 221
Postoperative Hospital Stay (day)	27 ± 24	22 ± 23
Mortality	1	1
Postoperative Complications	10/37 (27%)	31/174 (18%)
Infectious Complications	5/37 (14%)	20/174 (11%)
Pneumonia*	4/37 (11%)	4/174 (2%)

*P < 0.05 between elderly and control groups

### Patient characteristics and early postoperative outcomes after DG (Table 2)

There were 25 and 109 patients in the elderly and control groups, respectively. Average age was 88 ± 2 years in the elderly group and 66 ± 8 years in the control group. The ratio of female patients in the elderly group was greater than that in the control group (14/25 (56%) vs. 34/109 (32%), *P* < 0.05). The distribution of disease stage did not differ between the two groups. The reconstructive methods to restore enteric continuity, however, were different in the two groups; approximately two-thirds of patients (16/25 (64%)) underwent a Roux-en-Y (RY) reconstruction in the elderly group, whereas a Billroth-I (B-I) type reconstruction was performed in two-thirds of patients (72/109 (66%)) in the control group. Although the percentage of patients undergoing a D1+ (40% vs. 34%) and D2 (48% vs. 65%) LN dissection was not different in the two groups, percentage of D1 dissections was increased in the elderly group (12%) compared to the control group (1%, *P* < 0.05). The number of retrieved LNs, operation time, and intraoperative blood loss did not differ between the two groups. The postoperative hospital stay was greater in the elderly group (28 ± 26 vs. 20 ± 11, *P* < 0.05). One patient died of bleeding in the pulmonary alveoli next day after the surgery in the control group (Tables 2 and 3), but overall postoperative mortality did not differ between the two groups. Overall morbidity did not differ between the two groups (32% vs. 16%), and the same was true for infectious complications (12% vs. 7%). Two of the three infectious complications in the elderly group were pneumonias, while in the control group pneumonia was seen in only one of eight patients with infectious complications. The overall rate of postoperative pneumonia in the elderly group was greater than that in the control group (8% vs. 1%, *P* < 0.05). Two patients with pneumonia in the control group were Grade II in Clavien-Dindo classification, and treatments including antibiotics waere effective in these two patients. One patient who had pneumonia in the elderly group needed intensive care (Grade IV) but recovered (Table 3).

**Table 2:** Patient characteristics and perioperative results after DG

	Elderly (*N* = 25)	Control (*N* = 109)	Overall (*N* = 134)
Age (Years)*	82 ± 2	66 ± 8	69 ± 10
Male:Female*	11:14	75:34	86:48
Disease Stage			
I	12 (48%)	62 (57%)	74 (55%)
II	6 (24%)	16 (15%)	22 (17%)
III	7 (28%)	31 (28%)	38 (28%)
Reconstruction*			
B-I	9 (36%)	72 (66%)	81 (60%)
RY	16 (64%)	29 (27%)	45 (34%)
B-II	0	8 (7%)	8 (6%)
LN Dissection*			
D1	3 (12%)	1 (1%)	4 (3%)
D1+	10 (40%)	37 (34%)	47 (35%)
D2	12 (48%)	71 (65%)	83 (62%)
Number of retrieved LNs	27 ± 11	29 ± 15	28 ± 14
Operation Time (min)	228 ± 47	243 ± 49	240 ± 49
Blood Loss (mL)	231 ± 182	289 ± 201	278 ± 200
Postoperative Hospital Stay (day)*	28 ± 26	20 ± 11	22 ± 15
Mortality	0	1	1
Postoperative Complications	8/25 (32%)	17/109 (16%)	25/134 (19%)
Infectious Complications	3/25 (12%)	8/109 (7%)	11/134 (8%)
Pneumonia*	2/25 (8%)	1/109 (1%)	3/134 (2%)

*B-I* Billroth-I, *RY* Roux-en-Y, *B-II* Billroth-II, *LN* lymph node

*P < 0.05 between elderly and control groups

**Table 3:** Number of patients with postoperative complications in each group according to Clavien-Dindo classification

	DG				TG			
	Elderly		Control		Elderly		Control	
Grade I	4		24		1		10	
Grade II	7	8*	14	17*	1	2*	12	14*
Grade III	–		1		–		2	
Grade IV	1		1		–		–	
Grade V	–		1		1		–	

*Number of patients for Grade II-V

### Patient characteristics and early postoperative outcomes after TG (Table 4)

There were 12 and 65 patients in the elderly and the control groups, respectively. The average age was 84 ± 3 years in the elderly group and 66 ± 10 years in the control group. The percentage of female patients in the elderly group was greater than that in the control group (7/12 (58%) vs. 17/65 (26%), *P* < 0.05), as seen in patients with DG. There were no differences between groups in the distribution of disease stage, method of reconstruction, degree of LN dissection, number of retrieved LNs, operation time, intraoperative blood loss, and postoperative hospital stay. One patient died of pneumonia in the elderly group, and mortality in the elderly group was greater than that in the control group (1/12 vs 0/65, *P* < 0.05). Pneumonia was identified in two patients, and we could not find any other postoperative complications in the elderly group. Overall morbidity (17% vs. 22%) and infectious complications (17% vs. 18%) did not appear different between the two groups. The rate of pneumonia in the elderly group (17%) was greater than that in the control group (5%, *P* < 0.05). Antibiotics were effective in three and one patients with pneumonia in the control and the elderly groups, respectively, and all of these patients were Grade II. One patient in the elderly group died of pneumonia as stated above.

**Table 4:** Patient characteristics and perioperative results after TG

	Elderly (*N* = 12)	Control (*N* = 65)	Overall (*N* = 77)
Age (Years)*	84 ± 3	66 ± 10	69 ± 11
Male:Female*	5:7	48:17	53:24
Disease Stage			
I	7 (58%)	26 (40%)	33 (43%)
II	0	13 (20%)	13 (17%)
III	5 (42%)	26 (40%)	31 (40%)
Reconstruction			
RY	12 (100%)	63 (97%)	75 (97%)
Pouch	0	2 (3%)	2 (3%)
LN Dissection			
D1	0	2 (3%)	2 (3%)
D1+	10 (83%)	48 (74%)	58 (75%)
D2	2 (17%)	15 (23%)	17 (22%)
Number of retrieved LNs	23 ± 15	28 ± 13	27 ± 13
Operation Time (min)	241 ± 15	261 ± 54	258 ± 53
Blood Loss (mL)	367 ± 399	346 ± 248	349 ± 273
Postoperative Hospital Stay (day)	24 ± 20	26 ± 34	26 ± 32
Mortality*	1	0	1
Postoperative Complications	2/12 (17%)	14/65 (22%)	16/77 (21%)
Infectious Complications	2/12 (17%)	12/65 (18%)	14/77 (18%)**
Pneumonia	2/12 (17%)	3/65 (5%)	5/77 (6%)

*RY* Roux-en-Y, *LN* lymph node

* *P* < 0.05 between elderly and control groups, ** *P* < 0.05 compared to DG

The percentage of infectious complications after DG was less than that after TG (11/134 (8%) vs. 14/77 (18%), *P* < 0.05), but overall complications (25/134 (19%) vs. 16/77 (21%)) and pneumonia (3/134 (2%) vs. 5/77 (6%)) did not differ between DG and TG (Tables 2 and 4).

## Discussion

In the present study, we found that postoperative pneumonia (8%) in elderly patients was increased compared to the younger control group (1%) after DG, but not after TG. In general, TG has been thought to be a risk factor for postoperative pneumonia [11, 17], and the incidence of postoperative pneumonia after TG is generally considered greater than that after DG. In patients after TG, jejunal contents easily regurgitates into the esophagus because of the resection of the lower esophageal sphincter (LES) which plays an important role in prevention of gastroesophageal reflux [18]; after DG, however, LES is preserved. Airway inflammation and pneumonia are well known to increase with age [19, 20] due to swallowing and immune dysfunctions [21, 22]. Another factor of aging that may increase pneumonia is the well described age-related dysfunction of the LES [23]. In patients after DG, the increased incidence of pneumonia in elderly patients is likely to be enhanced compared to control patients because of generally low incidence of pneumonia and all these age-related aggravating factors (swallowing, immune regulation, and LES dysfunctions). While in patients after TG, the increase of pneumonia in elderly patients is likely to be unclear compared to control patients because of generally high incidence of pneumonia and lack of age-related LES dysfunction as an aggravating factor. These facts may be able to explain some of results of the present study. We also found that 4 of 5 infectious complications in elderly patients after gastrectomy were pneumonia, emphasizing the importance of pulmonary care in the elderly.

The present study compared overall morbidities and postoperative pneumonia between elderly and control patients separately after DG and TG, which has been rarely investigated in previous studies. A considerable number of articles has concluded that overall morbidity as well as pneumonia in elderly patients did not differ from those in young patients [3, 7–10]. In contrast, a couple of studies reported increased overall morbidity and pneumonia in elderly patients [5, 24]. Overall complications but not pneumonia were increased in elderly patients in another report [6]. Although one report identified age, pulmonary function, diabetes, and blood transfusion as risk factors for postoperative pneumonia [13], age was not a risk factor for pneumonia or morbidity in other studies [11, 12, 25]. Thus, the reported results are still controversial in terms of the incidence of postoperative pneumonia in elderly patients. It is likely that the elderly patients undergoing a gastrectomy were in with good general condition and thus were a selected population, while gastrectomy may have been avoided in those elderly patients at high risk secondary to their comorbidities. Different criteria to perform gastrectomy in each institution might have caused these different results. Pneumonia might have been increased in institutions where gastrectomy was performed in elderly patients whose general condition was relatively poor. Another possible reason for these different results is definition of elderly patients. We defined patients 80 years of age and above as ‘elderly.’ Most studies used the same criterion as ours [5–8], but others defined the elderly as > 75 [3, 9, 10, 25], 70 [26], and > 65 [27]. Maybe we should take into account age distribution of patients with gastric cancer and mean life span in each country before defining ‘elderly’ patient.

The rate of infectious postoperative complications in TG (18%) was greater than DG (8%) in the present study, although overall postoperative morbidity did not differ between DG (19%) and TG (21%). Similarly, no differences were observed in overall postoperative complications between DG and TG after open [28] and laparoscopic [29] gastrectomy. The results of a report by Bozzetti et al. [28] suggested that infectious complications after TG tended to be greater than those after DG. The rate of anastomotic leakage as well as major postoperative complications was greater after TG than after DG in laparoscopic surgery [30]. These results in previous studies are consistent with our results. We have to consider the definition of morbidity (Clavien-Dindo classification) and the operative approach (laparoscopic or open gastrectomy) in interpreting results on postoperative complications after gastrectomy.

The percentage of female patients was greater in elderly patients in our study. Similar results were found in a previous study of patients with gastric cancer > 85 years old compared to those 75–84 years of age [31]. The ratio of males to females with gastric cancer was the least at the age of 35, increased with age, peaked at the age of 60, decreased up to the age of 75, and remained around that level at the age of 80–85 [32].

We performed a RY procedure as the reconstructive method of choice after DG in most of elderly patients, because of presumed lesser incidence of anastomotic leakage in RY reconstruction compared to a B-I type reconstruction [33]; anastomotic leakage might be crucial especially in elderly patients.

It is a limitation of this study that this is a retrospective study with relatively small number of patients, and this could be a confounding factor for early postoperative outcomes.

## Conclusions

Overall morbidity rate did not appear to differ between elderly and control patients subjected to DG and TG. Although pneumonia in elderly patients appeared to be greater than that in younger patients after DG, a similar phenomenon was not observed after TG. This observation may be due to a different mechanism of postoperative pneumonia after DG vs. TG.

## Data Availability

The datasets generated and/or analyzed during the current study are available from the corresponding author on reasonable request.

## Abbreviations

B-I: Billroth-I
DG: Distal gastrectomy
LES: Lower esophageal sphincter
LN: Lymph node
RY: Roux-en-Y
TG: Total gastrectomy
